# n-3 PUFA-supplemented perioperative immunonutrition on postoperative outcomes in gastric cancer: a systematic review and meta-analysis

**DOI:** 10.3389/fnut.2025.1717989

**Published:** 2026-01-12

**Authors:** Fan Yang, Bei Zhou, Lebin Yang, Zheng Wei, Yi Liang, Xuan Wang, Hanguo Shan

**Affiliations:** 1 The Second Affiliated Hospital, Hengyang Medical School, University of South China, Hengyang, Hunan, China; 2 The Second Affiliated Hospital, Gastrointestinal Surgery, Hengyang Medical School, University of South China, Hengyang, Hunan, China

**Keywords:** gastric cancer, immunotrition, n-3 PUFA, perioperative, postoperative outcomes

## Abstract

**Objective:**

To evaluate the impact of perioperative immunonutritional regimens that included n-3 polyunsaturated fatty acids (n-3 PUFAs)—ranging from n-3 PUFA monotherapy to complex formulas combined with other immunonutrients—on postoperative outcomes in gastric cancer patients.

**Background:**

Although preventing postoperative complications is crucial for gastric cancer patients, consensus is lacking on the efficacy and optimal timing of administration of perioperative immunonutrition supplemented with n-3 PUFAs.

**Methods:**

We systematically searched PubMed, Embase, Web of Science, and the Cochrane Library for randomized controlled trials (RCTs) evaluating perioperative immunonutrition with n-3 PUFAs in gastric cancer surgery patients. Manual searches of reference lists were also conducted. The primary endpoint was total postoperative complications. Secondary endpoints included immune function [CD4+ T lymphocytes (CD4+ cells), CD8+ T lymphocytes (CD8+ cells), CD4+/CD8+ ratio, total lymphocytes, immunoglobulins IgA, IgG, IgM], nutritional status (transferrin, albumin, prealbumin), inflammatory markers (IL-6, TNF-α, CRP), and recovery indices (time to first flatus, length of hospital stay). Data were analyzed using RevMan v5.3 with a random-effect model.

**Results:**

Sixteen RCTs involving 1,642 patients were included. The meta-analysis demonstrated that perioperative n-3 PUFA supplementation significantly reduced the overall incidence of postoperative complications and promoted earlier recovery, as evidenced by a shortened time to first flatus and a reduced length of hospital stay. Furthermore, the n-3 PUFA group showed significant improvements in immunological (CD4+ T cell (%), CD4+/CD8+ ratio, total lymphocytes, IgA, IgG), inflammatory (IL-6, TNF-α, CRP), and nutritional (prealbumin) parameters. However, no significant differences were observed in CD8+ T cell (%), IgM, transferrin, or albumin levels between groups.

**Conclusion:**

The perioperative application of immunonutrition containing n-3 PUFAs can reduce the incidence of postoperative complications in patients with gastric cancer, improve immune function and nutritional status, mitigate inflammatory responses, and promote early postoperative recovery.

## Background

According to the latest GLOBOCAN report, gastric cancer ranks fifth in global cancer incidence, with a projected 62% surge in new cases by 2040 (1, 2). Surgical resection, the primary treatment, often induces a hypermetabolic state and negative nitrogen balance, leading to malnutrition and increased risk of postoperative complications (3, 4). This malnutrition-associated immune dysfunction further compromises tolerance to adjuvant therapies and overall treatment outcomes.

Immunonutrition, which supplements standard formulas with specific immunomodulatory nutrients such as arginine, glutamine, and n-3 polyunsaturated fatty acids (PUFAs), has been developed to counter these challenges (5). These components function through distinct yet complementary pathways: arginine supports T-cell function and vascular tone via nitric oxide synthesis (6), while glutamine serves as a primary fuel for immune cells and enterocytes, maintaining intestinal barrier integrity (7).

Notably, n-3 PUFAs—eicosapentaenoic acid (EPA) and docosahexaenoic acid (DHA)—play a unique and pivotal role. As highlighted by Calder (8), upon incorporation into cell membranes, n-3 PUFAs not only competitively inhibit pro-inflammatory eicosanoid synthesis but are also metabolized into specialized pro-resolving mediators (SPMs), such as resolvins and protectins. These SPMs actively promote the resolution of inflammation, going beyond mere suppression to facilitate tissue repair and a return to homeostasis (8). This dual mechanism makes n-3 PUFAs particularly valuable in modulating the sustained inflammatory response following surgery.

Despite the widespread clinical use of immunonutrition containing n-3 PUFAs, consensus is lacking regarding their efficacy and optimal timing in elective gastric cancer surgery (9, 10). Therefore, this systematic review and meta-analysis aims to evaluate the impact of perioperative immunonutrition containing n-3 PUFAs on postoperative outcomes in patients undergoing gastric cancer surgery.

## Methods

### Standard protocol approval, registration, and patient consent

Our research program has been registered with PROSPERO, an international systematic review registration website (registration number: CRD420251119214). The study was conducted and reported in accordance with the Cochrane Handbook, the Preferred Reporting Items for Systematic Reviews and Meta-Analyses (PRISMA) 2020 Statement (11). Owing to the nature of this study, informed consent and institutional review board approval were not needed.

### Search strategy

**Figure 1:**
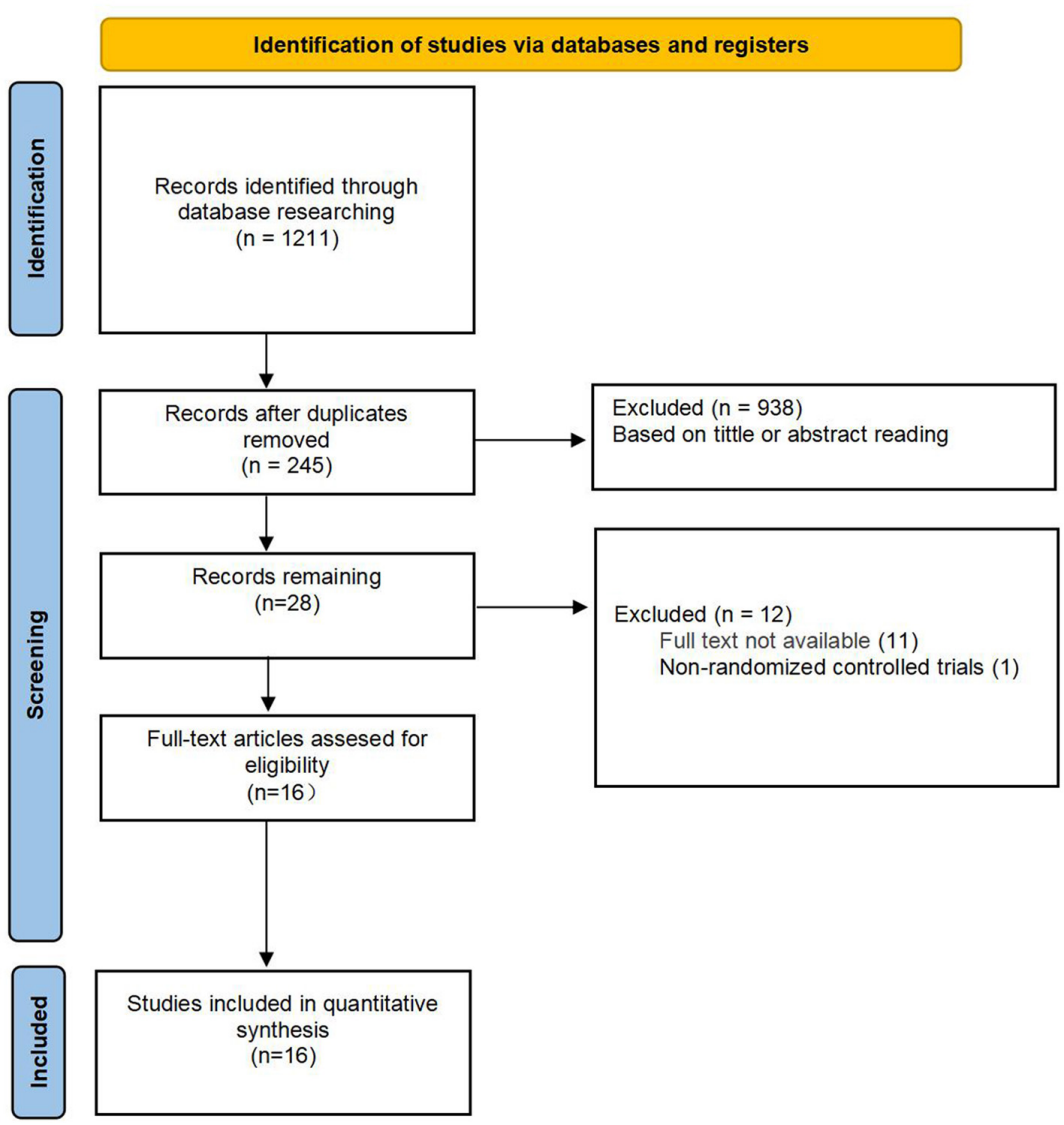
Flowchart of study selection.

We systematically searched PubMed, Embase, Web of Science, and the Cochrane Library for randomized controlled trials related to the influence of perioperative immunonutrition containing n-3 PUFAs in patients following gastric cancer surgery from January 1, 2000, until August 1, 2025. We used free text terms related to gastric cancer, n-3 polyunsaturated fatty acids (including synonyms) and closely related words such as “Stomach Neoplasm,” “Gastric Neoplasms,” “Neoplasms, Stomach,” “Cancer of Stomach,” “Stomach Cancers” and “Acid, N-3 Fatty,” “Fatty Acid, N-3,” “N 3 Fatty Acid,” “Omega-3 Fatty Acid,” “Acid, Omega-3 Fatty,” and “Fatty Acid, Omega-3” for our search. Additionally, we used Medical Subject Headings (MeSH) to search related literature in PubMed, Embase, Web of Science and the Cochrane Library with keywords such as “Neoplasm,” “Stomach” and “N-3 Fatty Acid.” Finally, we conducted the search by combining the two approaches (Supplementary Table 1). We used Zotero software to import titles and abstracts and initially screened for duplicates and citations that did not meet the inclusion criteria. Researchers subsequently independently reviewed the full texts of the preliminarily screened studies to assess whether they met the inclusion criteria. Any discrepancies between the two screenings were resolved by a third-party evaluator. A flowchart detailing the screening process is provided in Figure 1.

### Inclusion and exclusion criteria

The selection criteria for this study adhered to the PICOS principles (population, intervention, comparison, outcome, and study design).

**Inclusion criteria:**

(1) Population: Patients who underwent gastrectomy
(2) Intervention: Perioperative immunonutritional therapy incorporating n-3 PUFAs
(3) Comparison: standard nutritional therapy
(4) Results: Occurrence of postoperative infectious complications, postoperative immune function, nutritional status, and early recovery-related indicators
(5) Study design: Randomized controlled trials

**Exclusion criteria:**

(1) Study subjects were non-postgastrectomy patients for gastric cancer
(2) Full text of the study was unavailable
(3) Case reports, reviews, expert opinions, meta-analyses, and conference reports
(4) Non-randomized controlled trials

### Data extraction

The researchers utilized a predesigned data extraction table to manually extract data using Excel software. Any discrepancies between the two researchers were resolved by discussion or by a third-party decision. The following characteristics were independently extracted by the researchers: first author, year of publication, region, intervention method, sample size, age, sex, intervention duration, and the presence or absence of arginine.

#### Assessment of risk of bias

**Figure 2:**
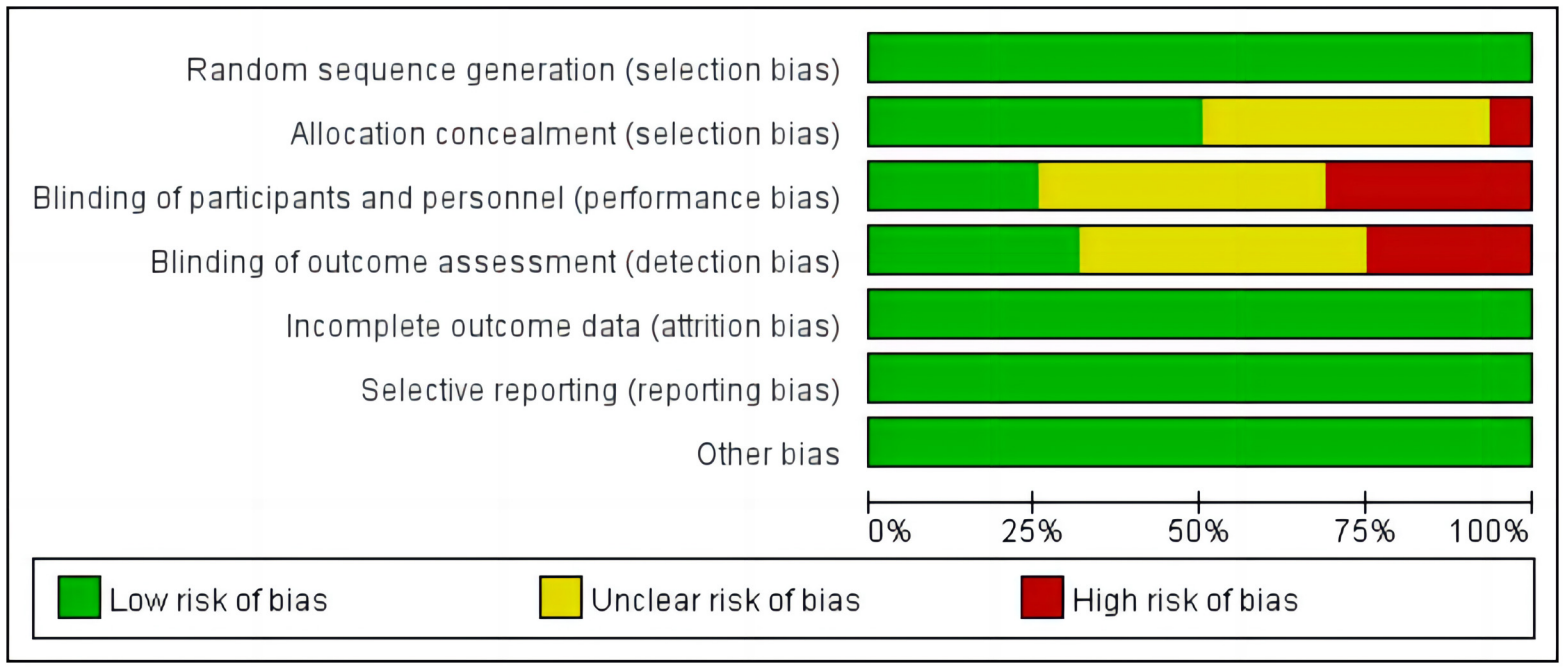
Risk of bias graph of the included studies based on the Cochrane collaboration tool.

**Figure 3:**
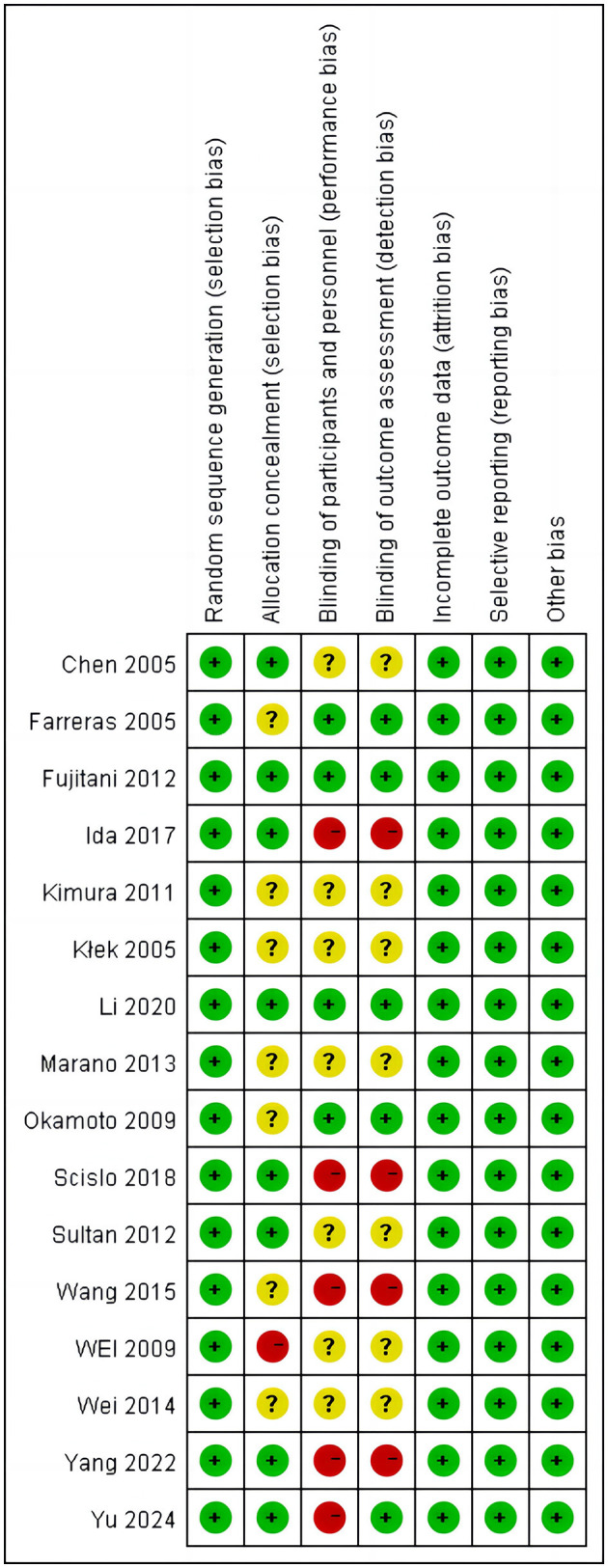
Risk of bias summary of the included studies based on the Cochrane collaboration tool.

Each included study was independently evaluated with the Cochrane collaboration tool (12); Figures 2, 3 and Table 1 summarize the risk of bias (ROBS) of the included studies. The ROBs for incomplete outcome data, selective reporting, random sequence generation, and other types of bias were low. The ROBs for participant and personnel blinding, outcome assessment blinding, and allocation concealment were classified as low, “some concerns” or high, respectively.

**Table 1:** Summary of risk of bias assessment for included studies (*n* = 16).

**Domain**	**Low risk**	**Some concern**	**High risk**
Random sequence generation	16	0	0
Allocation concealment	8	7	1
Blinding of participants and personnel	4	7	5
Blinding of outcome assessment	5	7	4
Incomplete outcome data	16	16	16
Selective reporting	16	16	16
Other bias	16	16	16

#### Publication bias

Regarding postoperative complications, as shown in Supplementary eFigure 2, the funnel plot for total postoperative complications was symmetrical, indicating minimal publication bias in its reporting. Per the Cochrane Handbook, funnel plots were not prepared for any other secondary outcome measures because fewer than 10 references were identified for each of these outcomes.

#### Assessing the strength of evidence

The Grading of Recommendations Assessment, Development and Evaluation (GRADE) system was used to grade the strength of evidence in identified associations. As a scientifically rigorous and transparent method developed by the GRADE working group, it is applied to assess the quality of evidence and grade the strength of recommendations in clinical practice guidelines. This assessment incorporates five factors that downgrade the evidence level: risk of bias, publication bias, imprecision, inconsistency, and indirectness. The evidence classification used in this study is presented in Table 2.

**Table 2:** Grading evidence based on the GRADE grading system.

**Outcomes**	**RR or MD (95%CI)**	**No of studies (participants)**	**Risk of bias**	**Imprecision**	**Inconsistency**	**Indirectness**	**Publication bias**	**Quality of evidence**
Total postoperative complications	0.74 (0.57, 0.95)	14 (1,508)	⊕	⊕	⊕	⊕	⊕	High-quality
CD4+ T cells	6.22 (5.11, 7.32)	5 (370)	⊕	⊕	⊕	⊕	○	Moderate quality
CD4/CD8 ratio	0.24 (0.08, 0.40)	5 (306)	⊕	⊕	⊕	⊕	○	Moderate quality
CD8+ T cells	−0.08 (−2.11, 1.96)	4 (246)	⊕	⊕	⊕	⊕	○	Moderate quality
Total lymphocytes	0.18 (0.06, 0.30)	4 (278)	⊕	⊕	⊕	⊕	○	Moderate quality
IgA	0.38 (0.24, 0.53)	4 (316)	⊕	⊕	⊕	⊕	○	Moderate quality
IgG	0.82 (0.40, 1.24)	3 (192)	⊕	⊕	⊕	⊕	○	Moderate quality
IgM	0.08 (−0.01, 0.07)	3 (192)	⊕	⊕	⊕	⊕	○	Moderate quality
Transferrin	0.05 (−0.02, 0.13)	7 (539)	⊕	⊕	⊕	⊕	○	Moderate quality
Albumin	1.76 (0.03, 3.50)	6 (430)	⊕	⊕	⊕	○	○	Low quality
Prealbumin	18.90 (7.17, 30.64)	4 (264)	⊕	⊕	⊕	⊕	○	Moderate quality
IL-6	−26.93 (−33.37, −20.49)	4 (245)	⊕	⊕	⊕	⊕	○	Moderate quality
TNF-α	−14.06 (−21.55, −6.56)	3 (133)	⊕	⊕	⊕	⊕	○	Moderate quality
CRP	−21.52 (−22.71, −20.33)	3 (291)	⊕	⊕	⊕	⊕	○	Moderate quality
Time to first exhaust	−9.01 (−14.85, −3.17)	3 (207)	⊕	⊕	⊕	⊕	○	Moderate quality
Length of hospital stay	−2.36 (−3.77, −0.96)	3 (216)	⊕	⊕	⊕	⊕	○	Moderate quality

⊕ = No downgrade; ○ = Downgraded by one level.

### Statistical analysis

Statistical analyses were performed using Review Manager 5.3 software. For categorical data, the relative risk (RR) and its 95% confidence interval (CI) were used for analysis. For continuous data, the mean difference (MD) and its 95% confidence interval (CI) were calculated. Heterogeneity among studies was assessed using the I^2^ statistic. Given the inherent clinical heterogeneity in nutritional interventions—such as variations in specific nutrient composition, administration routes, and intervention timing—a random-effects model was deemed the most appropriate a priori for all meta-analyses, as it provides a more conservative and generalizable estimate under these conditions. To ensure robustness, we also conducted analyses using a fixed-effect model for outcomes with low statistical heterogeneity (*I*^2^ < 25%). The results from both models were compared, and no material differences were found; therefore, the random-effects model results are presented throughout for consistency. Funnel plots were used to evaluate publication bias in the studies. A symmetric distribution of the funnel plot indicated no significant publication bias in the meta-analysis results, whereas an asymmetric distribution suggested the existence of publication bias. If the number of included studies was less than 10, publication bias was not analyzed. To elucidate the influence of effect modifiers, subgroup analyses according to intervention timing (preoperative, postoperative, or perioperative), intervention method (enteral immunonutrition, parenteral immunonutrition), duration of postoperative administration (< 7 days or ≥7 days), and difference in nutrient composition (n-3 PUFAs alone, arginine + n-3 PUFAs + RNA, and arginine + n-3 PUFAs + glutamine) were performed when sufficient data were available. Owing to insufficient data from relevant studies, subgroup analysis based on preoperative medication timing could not be performed.

## Results

### Selection of the included studies

In the initial search, a total of 1,211 articles were retrieved, 245 of which were excluded because they were duplicate records. After screening, 966 articles were initially identified, and 938 were eliminated on the basis of title and abstract review. Of the 28 articles that underwent full-text review, 12 were excluded because the full text was not available or because they were non-randomized controlled trials. Ultimately, 16 studies were included in this meta-analysis (Figure 1).

### Study characteristics

The baseline characteristics of the patients included in the studies are shown in Table 3. All the included studies were published between 2000 and 2024. The analysis included 16 studies (13–28) with a total sample size of 1,642 patients. The analysis involved studies from 6 countries across two continents: 11 (14, 16, 18–22, 25–28) from Asia and five (13, 15, 17, 23, 24) from Europe. With respect to nutritional intervention timing, four studies (21, 25, 26, 28) were conducted preoperatively, nine were conducted postoperatively (13–16, 20, 22–24, 27), and 3 were conducted perioperatively (17–19). Moreover, 9 studies (13, 16, 21–26, 28) included arginine, and 7 studies (14, 15, 17–20, 27) did not.

**Table 3:** Characteristics of the studies included in the meta-analysis.

**First author**	**Year**	**Region**	**Intervention method**	**Sample size**	**Age (mean)**	**Male (%)**	**Intervention duration**	**Nutrient composition**
Scislo et al.	2018	Poland	Enteral immunonutrition	99	62.9	72.5	Post: 6 days	arginine, glutamines and omega-3 fatty acids
WEI et al.	2009	China	Parenteral immunonutrition	40	71.9	70.0	Post: 5 days	omega-3 fatty acids
Chen et al.	2005	China	Enteral immunonutrition	40	59.0	70.0	Post: 9 days	arginine, glutamines and omega-3 fatty acids
Wang et al.	2015	China	Parenteral immunonutrition	47	60.0	74.5	Pre: 7 days Post: 6 days	omega-3 fatty acids
Sultan et al.	2012	UK	Enteral immunonutrition	132	67.0	75.8	Pre: 7 days Post: 7 days	omega-3 fatty acids
Ida et al.	2017	Japan	Enteral immunonutrition	123	65.0	72.4	Pre: 7 days Post: 21 days	omega-3 fatty acids
Yang et al.	2022	China	Parenteral immunonutrition	120	57.8	68.3	Post: 5 days	omega-3 fatty acids
Yu et al.	2024	China	Enteral immunonutrition	112	61.7	73.2	Pre: 7 days	arginine, nucleotides and omega-3 fatty acids
Li et al.	2020	China	Enteral immunonutrition	124	56.2	56.5	Post: 5 days	arginine, glutamines and omega-3 fatty acids
Farreras et al.	2005	Spain	Enteral immunonutrition	60	68.0	53.3	Post: 7 days	arginine, nucleotides and omega-3 fatty acids
Marano et al.	2013	Italy	Enteral immunonutrition	109	65.8	65.1	Post: 7 days	arginine, nucleotides and omega-3 fatty acids
Okamoto et al.	2009	Japan	Enteral immunonutrition	60	68.9	70.0	Pre: 7 days	arginine, nucleotides and omega-3 fatty acids
Kimura et al.	2011	Japan	Enteral immunonutrition	240	64.8	75.0	Pre: 5 days	arginine, nucleotides and omega-3 fatty acids
Kłek et al.	2005	Poland	Parenteral immunonutrition	60	61.9	56.7	Post: 7 days	omega-3 fatty acids
Wei et al.	2014	China	Parenteral immunonutrition	46	54.2	56.5	Post: 6 days	omega-3 fatty acids
Fujitani et al.	2012	Japan	Enteral immunonutrition	231	64.0	72.4	Pre: 5 days	arginine, nucleotides and omega-3 fatty acids

### Meta-analysis results

#### Total postoperative complications

A total of 14 studies (13–15, 17–21, 23–28) reported total postoperative complications. The results of meta-analysis demonstrated that compared with the standard nutrition group, the immunonutrition group had a lower incidence of postoperative complications (RR, 0.74; 95% CI, 0.57–0.95; *p* = 0.02), with substantial heterogeneity (*I*^2^ = 57%). A targeted sensitivity analysis was performed to investigate the sources of this heterogeneity. The studies by Sultan et al. (17) and Fujitani et al. (26) were identified as potential outliers due to distinct clinical characteristics. When these two studies were excluded, the pooled result remained significant and the heterogeneity was substantially reduced (RR, 0.63; 95% CI, 0.47–0.84; *p* = 0.002; *I*^2^ = 35%). We postulate that the unique populations—Sultan et al. enrolled patients with esophagogastric junction cancers, and Fujitani et al. evaluated an exclusive preoperative regimen—are key clinical drivers of the initial heterogeneity. The certainty of the evidence was deemed high. A forest plot of total postoperative complications is shown in Supplementary eFigure 1..

Subgroup analysis for differences in intervention timing revealed significantly fewer complications in the postoperative group than in the preoperative group and the perioperative group (*p* = 0.007; Supplementary eFigure 18), and the subgroup analysis revealed no differences in efficacy between the groups according to type of immunonutrition regimen (*p* = 0.76; Supplementary eFigure 19), specific immunonutrient composition (*p* = 0.91; Supplementary eFigure 20), duration of postoperative administration (*p* = 0.42; Supplementary eFigure 21), or intervention method (*p* = 0.07; Supplementary eFigure 24).

#### CD4+ T cell (%)

A total of five studies (14, 16, 20, 22, 27) reported on the percentage of CD4+ T cell. The results of the meta-analysis demonstrated that compared with the standard nutrition group, the immunonutrition group had higher levels of CD4+ T cell (MD = 6.22%; 95% CI, 5.17–7.32; *p* < 0.00001; *I*^2^ = 16%). The evidence level was downgraded to moderate due to the limited number of studies (< 10), prompting concerns of publication bias. A forest plot of CD4+ T cell (%) is shown in Supplementary eFigure 3.

#### CD4/CD8 ratio

A total of five studies (14, 16, 20, 25, 27) reported the CD4/CD8 ratio. The results of the meta-analysis demonstrated that compared with the standard nutrition group, the immunonutrition group had higher CD4/CD8 ratios [MD = 0.24; 95% CI, (0.08–0.40); *p* = 0.003; *I*^2^ = 25%]. The evidence level was downgraded to moderate due to the limited number of studies (< 10), prompting concerns of publication bias. A forest plot of the CD4/CD8 ratio is shown in Supplementary eFigure 4.

#### CD8+ T cell (%)

A total of four studies (14, 16, 20, 27) reported data on the percentage of CD8+ T cell. The results of the meta-analysis revealed no significant difference in CD8+ T cell (%) between the immunonutrition group and the standard nutrition group [MD = −0.08%; 95% CI, (−2.11 to 1.96); *p* = 0.94; *I*^2^ = 18%]. The evidence level was downgraded to moderate due to the limited number of studies (< 10), prompting concerns of publication bias. A forest plot of the CD8+ T cell (%) is shown in Supplementary eFigure 5.

#### Total lymphocytes

A total of four studies (21, 23, 25, 27) reported total lymphocyte counts. The results of the meta-analysis demonstrated that compared with the standard nutrition group, the immunonutrition group had greater total lymphocyte counts (MD = 0.18 × 10^9^/L; 95% CI, 0.06–0.30; *p* = 0.004; *I*^2^ = 35%). The evidence level was downgraded to moderate due to the limited number of studies (< 10), prompting concerns of publication bias. A forest plot of the total lymphocyte is shown in Supplementary eFigure 6.

#### IgA (g/L)

A total of four studies (14, 16, 21, 22) reported IgA levels. The results of the meta-analysis demonstrated that compared with the standard nutrition group, the immunonutrition group had higher IgA levels [MD = 0.38 g/L; 95% CI, (0.24–0.53); *p* < 0.00001; *I*^2^ = 0%]. The evidence level was downgraded to moderate due to the limited number of studies (< 10), prompting concerns of publication bias. A forest plot of the IgA is shown in Supplementary eFigure 7.

#### IgG (g/L)

A total of three studies (14, 16, 21) reported IgG levels. The results of the meta-analysis demonstrated that compared with the standard nutrition group, the immunonutrition group had higher IgG levels [MD = 0.82 g/L; 95% CI, (0.40–1.24); *p* = 0.0001; *I*^2^ = 0]. The evidence level was downgraded to moderate due to the limited number of studies (< 10), prompting concerns of publication bias. A forest plot of the IgG is shown in Supplementary eFigure 8.

#### IgM (g/L)

A total of three studies (14, 16, 21) reported IgM levels. The results of the meta-analysis revealed no significant difference in IgM levels between the immunonutrition group and the standard nutrition group [MD = 0.08 g/L; 95% CI, (−0.01 to 0.17); *p* = 0.08; *I*^2^ = 15%]. The evidence level was downgraded to moderate due to the limited number of studies (< 10), prompting concerns of publication bias. A forest plot of the IgM is shown in Supplementary eFigure 9.

#### Transferrin (g/L)

A total of seven studies (14, 16, 20, 22–24, 27) reported transferrin levels. The results of the meta-analysis revealed no significant difference in transferrin levels between the immunonutrition group and the standard nutrition group [MD = 0.05 g/L; 95% CI, (−0.02 to 0.13); *p* = 0.13; *I*^2^ = 37%]. The evidence level was downgraded to moderate due to the limited number of studies (< 10), prompting concerns of publication bias. A forest plot of the transferrin is shown in Supplementary eFigure 10.

#### Albumin (g/L)

A total of six studies (14, 16, 21–23, 27) reported albumin levels. The results of the meta-analysis demonstrated no significant difference in albumin levels between the immunonutrition group and the standard nutrition group [MD = 1.76 g/L; 95% CI, (0.03–3.50); *p* = 0.05], with considerable heterogeneity (*I*^2^ = 77%). A targeted sensitivity analysis was performed to investigate the sources of this heterogeneity. The study by Yang et al. (20) was identified as a potential outlier due to its distinct intervention protocol. When this study was excluded, the heterogeneity was substantially reduced (*I*^2^ = 33%), but the pooled result remained non-significant [MD = 0.90 g/L; 95% CI, (−0.33 to 2.13); *p* = 0.15]. We postulate that the unique design of Yang et al.—which employed a combination of enteral and parenteral immunonutrition, unlike the single-route protocols in other studies—is a key methodological driver of the initial heterogeneity. The level of evidence is low, owing to the small number of studies (< 10) with potential publication bias, and inconsistency (*I*^2^ = 77%). A forest plot of the transferrin is shown in Supplementary eFigure 11.

#### Prealbumin (mg/L)

A total of four studies (14, 16, 22, 23) reported prealbumin levels. The results of the meta-analysis demonstrated that prealbumin levels were greater in the immunonutrition group than in the standard nutrition group [MD = 18.90 mg/L; 95% CI, (7.17–30.64); *p* < 0.0001; *I*^2^ = 44%]. The evidence level was downgraded to moderate due to the limited number of studies (< 10), prompting concerns of publication bias. A forest plot of the prealbumin is shown in Supplementary eFigure 12.

#### IL-6 (pg/ml)

A total of four studies (16, 19, 21, 27) reported IL-6 levels. The results of the meta-analysis demonstrated that compared with the standard nutrition group, the immunonutrition group had lower IL-6 levels [MD = −26.93 pg/ml; 95% CI, (−33.37, −20.49); *p* < 0.00001; *I*^2^ = 45%]. The evidence level was downgraded to moderate due to the limited number of studies (< 10), prompting concerns of publication bias. A forest plot of the prealbumin is shown in Supplementary eFigure 13.

#### TNF-α (pg/ml)

Three studies (14, 19, 27) reported TNF-α levels. The results of the meta-analysis demonstrated that TNF-α levels were lower in the immunonutrition group than in the standard nutrition group [MD = −14.06 pg/ml; 95% CI, (−21.55, −6.56), *p* = 0.0002; *I*^2^ = 15%]. The evidence level was downgraded to moderate due to the limited number of studies (< 10), prompting concerns of publication bias. A forest plot of the TNF-α is shown in Supplementary eFigure 14.

#### CRP (mg/L)

Three studies (19, 20, 22) reported CRP levels. The results of the meta-analysis demonstrated that compared with the standard nutrition group, the immunonutrition group had lower CRP levels, as shown in Supplementary eFigure 15 [MD = −21.52 mg/L; 95% CI, (−22.71, −20.33), *p* < 0.00001; *I*^2^ = 0%]. The evidence level was downgraded to moderate due to the limited number of studies (< 10), prompting concerns of publication bias. A forest plot of the CRP is shown in Supplementary eFigure 15.

#### Time to first flatus (days)

A total of three studies (14, 19, 20) reported the time to first flatus. The results of the meta-analysis demonstrated that compared with the standard nutrition group, the immunonutrition group had a significantly shorter time to first flatus after surgery [MD = −9.01 days; 95% CI, (−14.85, −3.17); *p* = 0.002; *I*^2^ = 3%]. The evidence level was downgraded to moderate due to the limited number of studies (< 10), prompting concerns of publication bias. A forest plot of the time to first flatus is shown in Supplementary eFigure 16.

#### Length of hospital stay (days)

Three studies (19, 24, 25) reported the length of hospital stay. The results of the meta-analysis revealed that compared with the standard nutrition group, the immunonutrition group had a shorter hospital stay [MD = −2.36 days; 95% CI, (−3.77, −0.95), *p* = 0.001; *I*^2^ = 49%]. The evidence level was downgraded to moderate due to the limited number of studies (< 10), prompting concerns of publication bias. A forest plot of the length of hospital stay is shown in Supplementary eFigure 17.

## Discussion

### Summary of main findings

This meta-analysis of 16 randomized controlled trials (RCTs) demonstrates that perioperative immunonutrition supplemented with n-3 PUFAs significantly reduces the incidence of postoperative complications and accelerates early recovery, as evidenced by a shortened time to first flatus and length of hospital stay in patients undergoing gastrectomy for gastric cancer. Furthermore, it modulates systemic inflammatory responses and improves immune function and nutritional status. However, no significant improvements were observed in specific nutritional markers (transferrin, albumin) or certain immune parameters (CD8+ T-cell percentage, IgM).

### Clinical implications and the optimal timing of intervention

The observed clinical benefits transcend mere statistical significance. The approximate 2.5-day reduction in hospital stay aligns directly with the goals of Enhanced Recovery After Surgery (ERAS) protocols (29), facilitating a faster return to normal life, decreasing the risk of hospital-acquired infections, and potentially generating substantial healthcare cost savings. Furthermore, the 37% relative reduction in postoperative complications represents a major enhancement in patient safety by reducing morbidity, the need for re-intervention, and unplanned readmissions.

Critically, our subgroup analysis revealed that the significant reduction in complications was primarily driven by postoperative immunonutrition, which appeared more effective than preoperative or combined perioperative supplementation. This timing-specific benefit may be linked to the dynamics of the surgical stress response. The immediate postoperative period is characterized by a pronounced surge in pro-inflammatory cytokines and a state of systemic immune suppression (30). Administering n-3 PUFAs directly during this pro-inflammatory peak ensures their bioavailability for incorporation into cell membranes, allowing them to more effectively compete with arachidonic acid and serve as substrates for generating specialized pro-resolving mediators (SPMs) that actively counterregulate the inflammatory storm (31).

### Temporal evolution of surgical practice and generalizability

It is important to consider the temporal span of the included studies (2000–2024) in relation to evolving surgical standards. Over this period, minimally invasive techniques (e.g., laparoscopic and robotic gastrectomy) and Enhanced Recovery After Surgery (ERAS) protocols have been increasingly adopted, potentially reducing baseline surgical trauma and accelerating recovery. While these advancements may influence absolute outcome rates, the pathophysiological target of n-3 PUFA supplementation—namely, the modulation of postoperative systemic inflammation and immune dysfunction—remains relevant. The consistent benefit observed across studies, including more recent trials likely conducted within modern perioperative frameworks, supports the ongoing applicability of our findings. Nevertheless, the magnitude of effect should be interpreted in the context of contemporary multimodal care, and future trials explicitly designed within current surgical and ERAS settings are warranted to refine the precise role of immunonutrition in today's practice.

### Effects on immune and nutritional biomarkers and their clinical translation

#### Positive modulation of cellular immunity

Our analysis confirmed a significant beneficial effect of immunonutrition on key cellular immune parameters, specifically an increase in CD4+ T-cell counts and the CD4+/CD8+ ratio. This finding aligns with and reinforces the conclusion of a prior meta-analysis by Song et al. (40), which established a clear immune-modulatory benefit for patients undergoing gastric cancer surgery.

#### Reconciling immunological benefits with divergent clinical outcomes

However, a critical and instructive divergence emerges when examining clinical endpoints. Song et al. (40) reported no significant improvement in overall postoperative complications, infection rates, or length of hospital stay despite observing similar immune improvements. In contrast, our updated meta-analysis demonstrated a statistically significant reduction in total postoperative complications (RR, 0.74; 95% CI, 0.57–0.95). Several factors may explain this discrepancy and help reconcile the evidence across meta-analyses: (1) Expanded and Updated Evidence Base: our analysis incorporates 7 additional RCTs published after 2015. These newer trials may have been conducted within more optimized perioperative care frameworks (e.g., stricter ERAS protocols), where the adjunctive effect of immunonutrition on clinical recovery becomes more detectable; (2) Refined Analysis Addressing Heterogeneity: Song et al. (40) noted substantial heterogeneity in their clinical outcome analyses. In our work, a targeted sensitivity analysis that excluded outliers with distinct populations (e.g., Sultan et al. (17) and Fujitani et al. (26) yielded a more homogenous and robust estimate for complication reduction (RR, 0.63; 95% CI, 0.47–0.84; *p* = 0.002; *I*^2^ = 35%). This suggests that better accounting for clinical heterogeneity can clarify the true clinical effect size; (3) Formulation and Protocol Evolution: As highlighted in a subsequent network meta-analysis by Song et al. (41), the efficacy of immunonutrition may vary significantly depending on the specific formula used. The blend of studies in our updated analysis might proportionally contain more trials employing formulations or protocols with proven clinical efficacy.

#### Explanation for null findings on other biomarkers

In contrast to the positive findings on cellular immunity, no significant effect was observed for several other predefined immune and nutritional biomarkers, which warrants explanation. The non-response of transferrin and albumin, both negative acute-phase reactants, is likely due to their long half-lives (8–10 and 14–20 days, respectively); their serum concentrations are difficult to rapidly alter with short-term nutritional intervention, especially when the liver prioritizes the synthesis of positive acute-phase proteins like CRP in the perioperative period (37, 38).

Regarding immune cells and immunoglobulins, the null findings may be attributed to several factors: (1) the relatively small number of studies reporting CD8+ T cells (%) and IgM levels, resulting in limited statistical power; (2) the primary immunomodulatory action of n-3 PUFAs may not be a nonspecific boost in lymphocyte counts but a functional regulation of CD8+ T cells or alteration of macrophage polarization (8); (3) the early postoperative immunosuppressive state, coupled with the anti-inflammatory nature of n-3 PUFAs, may not favor a sharp increase in short-term IgM production (39); and (4) heterogeneity in immunonutrition formulas, routes of administration (enteral vs. parenteral), and assay methods across the included studies.

### Underlying mechanisms of immunonutrition

Immunonutrition, typically containing glutamine, arginine, n-3 PUFAs, and nucleotides, exerts its benefits through two primary pathways: ameliorating nutritional status and directly modulating immune function to enhance anti-infective capacity (6, 7). n-3 PUFAs, including EPA and DHA, are key mediators of the latter. They incorporate into cell membranes, influencing membrane fluidity and function. Through competitive inhibition, they reduce the production of pro-inflammatory eicosanoids from arachidonic acid and serve as precursors for potent SPMs like resolvins and protectins, which actively promote the resolution of inflammation (8, 31, 36). Furthermore, they can modulate the gene expression of inflammatory mediators and enhance the function of immune cells such as T and B lymphocytes (31, 33, 34). This multifaceted anti-inflammatory and pro-resolving action is crucial for mitigating the excessive postoperative inflammatory response triggered by surgical stress (6, 32, 35).

## Strengths and limitations

This review has several strengths. First, a comprehensive literature search across four major databases was conducted to minimize publication bias. Second, we adhered to PRISMA guidelines and rigorously assessed the quality of included RCTs using the Cochrane risk of bias tool. Third, we employed the GRADE methodology to assess the certainty of evidence for our primary outcomes, which provides a transparent and systematic framework for interpreting the reliability of our findings. Fourth, extensive subgroup analyses were performed to explore heterogeneity and provide evidence for optimal intervention strategies.

Several limitations must be acknowledged. First, the number of RCTs, particularly for specific biomarkers and recovery metrics, remains relatively small, necessitating further validation. Second, while subgroup analyses offered valuable insights, the sample sizes within these subgroups were relatively limited, potentially constraining the statistical power and precluding definitive conclusions, especially for the timing of intervention. Third, although we employed a random-effects model and conducted subgroup analyses based on nutrient composition, significant clinical heterogeneity persisted across studies regarding the specific immunonutrition formulations used (e.g., varying combinations of arginine, glutamine, RNA, and n-3 PUFAs, as well as differences in commercial products and dosages). This variability, while partly accounted for statistically, may still influence the interpretation and generalizability of the pooled effects. Fourth, the generalizability of our findings may be further limited as most studies were conducted in Asian populations. Fifth, some included trials reported very few events for the outcome of postoperative complications. The incorporation of such sparse event data can introduce instability and disproportionately influence the pooled effect size in meta-analyses. Although our primary conclusion is supported by the overall trend across a substantial number of patients (*n* = 1,642), the precision and robustness of the point estimate should be interpreted with this limitation in mind. Finally, while subgroup analyses were performed for the primary outcome (complication rate), they were lacking for secondary outcomes like immune indicators, and we could not identify the optimal preoperative intervention duration.

## Conclusion

In conclusion, this meta-analysis demonstrates that perioperative immunonutrition enriched with n-3 PUFAs is effective in reducing postoperative complications and accelerating recovery for patients undergoing gastrectomy. These benefits are likely mediated through the attenuation of systemic inflammation and immunomodulation.

While subgroup analyses suggest that the postoperative period might represent the most effective window for this nutritional intervention, this finding should be interpreted with caution due to the limited sample sizes within the subgroups, which precludes a definitive recommendation on timing.

Nevertheless, the overall efficacy supports the routine integration of n-3 PUFA-enriched immunonutrition into perioperative care protocols. Future large-scale, well-designed RCTs are warranted to confirm the optimal timing and duration of supplementation.
