# Supplementary material for: n-3 PUFA-supplemented perioperative immunonutrition on postoperative outcomes in gastric cancer: a systematic review and meta-analysis
Source: Front Nutr. 2026 Jan 12;12:1717989. doi: 10.3389/fnut.2025.1717989 (PMC12832536; doi:10.3389/fnut.2025.1717989)

**Supplemental materials**

1. General Search Strategies for PubMed, Embase, Web of science and Cochrane Library.
2. Forest Plot of total postoperative complications
3. Funnel Plot of total postoperative complications
4. Forest Plot of CD4+ T cells
5. Forest Plot of CD4/CD8 ratio
6. Forest Plot of CD8+ T cells
7. Forest Plot of total lymphocytes
8. Forest Plot of IgA
9. Forest Plot of IgG
10. Forest Plot of IgM
11. Forest Plot of transferrin
12. Forest Plot of albumin
13. Forest Plot of prealbumin
14. Forest Plot of IL-6
15. Forest Plot of TNF-α
16. Forest Plot of CRP
17. Forest Plot of time to first flatus
18. Forest Plot of length of hospital stay
19. Forest Plot for subgroup analysis of total postoperative complications according to intervention timing.
20. Forest Plot for subgroup analysis of total postoperative complications according to **type of immunonutrition regimen**.
21. Forest Plot for subgroup analysis of total postoperative complications according to specific immunonutrient composition.
22. Forest Plot for subgroup analysis of total postoperative complications according to duration of postoperative administration.
23. Forest Plot for subgroup analysis of total postoperative complications according to intervention method.

**eTable 1**. General Search Strategies for PubMed, Embase, Web of science and Cochrane Library.

Text S1 Search strategy

Database: Pubmed from inception to Present> (Search date: 01, 08, 2025)

Search Strategy:

1. "Stomach Neoplasms"[Mesh]
2. **(((((((((((((((((Neoplasm, Stomach[Title/Abstract]) OR (Stomach Neoplasm[Title/Abstract])) OR (Gastric Neoplasms[Title/Abstract])) OR (Gastric Neoplasm[Title/Abstract])) OR (Neoplasm, Gastric[Title/Abstract])) OR (Neoplasms, Gastric[Title/Abstract])) OR (Neoplasms, Stomach[Title/Abstract])) OR (Cancer of Stomach[Title/Abstract])) OR (Stomach Cancers[Title/Abstract])) OR (Cancer of the Stomach[Title/Abstract])) OR (Gastric Cancer[Title/Abstract])) OR (Cancer, Gastric[Title/Abstract])) OR (Cancers, Gastric[Title/Abstract])) OR (Gastric Cancers[Title/Abstract])) OR (Stomach Cancer[Title/Abstract])) OR (Cancers, Stomach[Title/Abstract])) OR (Cancer, Stomach[Title/Abstract])) OR (Gastric Cancer, Familial Diffuse[Title/Abstract])**
3. #1 OR #2
4. "Fatty Acids, Omega-3"[Mesh]
5. **(((((((((((((((((((((((((((((N-3 Fatty Acid[Title/Abstract]) OR (Acid, N-3 Fatty[Title/Abstract])) OR (Fatty Acid, N-3[Title/Abstract])) OR (N 3 Fatty Acid[Title/Abstract])) OR (Omega-3 Fatty Acid[Title/Abstract])) OR (Acid, Omega-3 Fatty[Title/Abstract])) OR (Fatty Acid, Omega-3[Title/Abstract])) OR (Omega 3 Fatty Acid[Title/Abstract])) OR (Omega-3 Fatty Acids[Title/Abstract])) OR (n-3 Oil[Title/Abstract])) OR (n 3 Oil[Title/Abstract])) OR (Oil, n-3[Title/Abstract])) OR (n3 Oil[Title/Abstract])) OR (Oil, n3[Title/Abstract])) OR (n-3 Fatty Acids[Title/Abstract])) OR (n 3 Fatty Acids[Title/Abstract])) OR (n-3 Polyunsaturated Fatty Acid[Title/Abstract])) OR (n 3 Polyunsaturated Fatty Acid[Title/Abstract])) OR (n-3 PUFA[Title/Abstract])) OR (n 3 PUFA[Title/Abstract])) OR (PUFA, n-3[Title/Abstract])) OR (n3 Fatty Acid[Title/Abstract])) OR (Fatty Acid, n3[Title/Abstract])) OR (n3 PUFA[Title/Abstract])) OR (PUFA, n3[Title/Abstract])) OR (n3 Polyunsaturated Fatty Acid[Title/Abstract])) OR (n3 Oils[Title/Abstract])) OR (Omega 3 Fatty Acids[Title/Abstract])) OR (n-3 Oils[Title/Abstract])) OR (n 3 Oils[Title/Abstract])**
6. #4 OR #5
7. #3 AND #6 (98 results)

Text S2 Search strategy

Database: EMBASE (Search date: 01, 08, 2025)

Search Strategy:

1. 'Stomach Neoplasms'/exp
2. ‘Neoplasm, Stomach’:ti,ab,kw OR ‘Stomach Neoplasm’:ti,ab,kw OR ‘Gastric Neoplasms’:ti,ab,kw OR ‘Gastric Neoplasm’:ti,ab,kw OR ‘Neoplasm, Gastric’:ti,ab,kw OR ‘Neoplasms, Gastric’:ti,ab,kw OR ‘Neoplasms, Stomach’:ti,ab,kw OR ‘Cancer of Stomach’:ti,ab,kw OR ‘Stomach Cancers’:ti,ab,kw OR ‘Cancer of the Stomach’:ti,ab,kw OR ‘Gastric Cancer’:ti,ab,kw OR ‘Cancer, Gastric’:ti,ab,kw OR ‘Cancers, Gastric’:ti,ab,kw OR ‘Gastric Cancers’:ti,ab,kw OR ‘Stomach Cancer’:ti,ab,kw OR ‘Cancers, Stomach’:ti,ab,kw OR ‘Cancer, Stomach’:ti,ab,kw OR ‘Gastric Cancer, Familial Diffuse’:ti,ab,kw
3. #1 OR #2
4. 'omega 3 fatty acid'/exp
5. ‘N-3 Fatty Acid’:ti,ab,kw OR ‘Acid, N-3 Fatty’:ti,ab,kw OR ‘Fatty Acid, N-3’:ti,ab,kw OR ‘N 3 Fatty Acid’:ti,ab,kw OR ‘Omega-3 Fatty Acid’:ti,ab,kw OR ‘Acid, Omega-3 Fatty’:ti,ab,kw OR ‘Fatty Acid, Omega-3’:ti,ab,kw OR ‘Omega 3 Fatty Acid’:ti,ab,kw OR ‘Omega-3 Fatty Acids’:ti,ab,kw OR ‘n-3 Oil’:ti,ab,kw OR ‘n 3 Oil’:ti,ab,kw OR ‘Oil, n-3’:ti,ab,kw OR ‘n3 Oil’:ti,ab,kw OR ‘Oil, n3’:ti,ab,kw OR ‘n-3 Fatty Acids’:ti,ab,kw OR ‘n 3 Fatty Acids’:ti,ab,kw OR ‘n-3 Polyunsaturated Fatty Acid’:ti,ab,kw OR ‘n 3 Polyunsaturated Fatty Acid’:ti,ab,kw OR ‘n-3 PUFA’:ti,ab,kw OR ‘n 3 PUFA’:ti,ab,kw OR ‘PUFA, n-3’:ti,ab,kw OR ‘n3 Fatty Acid’:ti,ab,kw OR ‘Fatty Acid, n3’:ti,ab,kw OR ‘n3 PUFA’:ti,ab,kw OR ‘PUFA, n3’:ti,ab,kw OR ‘n3 Polyunsaturated Fatty Acid’:ti,ab,kw OR ‘n3 Oils’:ti,ab,kw OR ‘Omega 3 Fatty Acids’:ti,ab,kw OR ‘n-3 Oils’:ti,ab,kw OR ‘n 3 Oils’:ti,ab,kw
6. #4 OR #5
7. #3 AND #6 (241 results)

Text S3 Search strategy

Database: Cochrane Library from inception to Present> (Search date: 01, 08, 2025)

Search Strategy:

1. MeSH descriptor: [Stomach Neoplasms] explode all trees
2. **(**Neoplasm, Stomach**)**:ti,ab,kw OR **(**Stomach Neoplasm):ti,ab,kw OR **(**Gastric Neoplasms):ti,ab,kw OR **(**Gastric Neoplasm):ti,ab,kw OR **(**Neoplasm, Gastric):ti,ab,kw OR **(**Neoplasms, Gastric):ti,ab,kw OR **(**Neoplasms, Stomach):ti,ab,kw OR **(**Cancer of Stomach):ti,ab,kw OR **(**Stomach Cancers):ti,ab,kw OR **(**Cancer of the Stomach):ti,ab,kw OR **(**Gastric Cancer):ti,ab,kw OR **(**Cancer, Gastric):ti,ab,kw OR **(**Cancers, Gastric):ti,ab,kw OR (Gastric Cancers):ti,ab,kw OR (Stomach Cancer):ti,ab,kw OR (Cancers, Stomach):ti,ab,kw OR (Cancer, Stomach):ti,ab,kw OR (Gastric Cancer, Familial Diffuse):ti,ab,kw
3. #1 OR #2
4. MeSH descriptor: [Fatty Acids, Omega-3] explode all trees
5. (N-3 Fatty Acid):ti,ab,kw OR (Acid, N-3 Fatty):ti,ab,kw OR (Fatty Acid, N-3):ti,ab,kw OR (N 3 Fatty Acid):ti,ab,kw OR (Omega-3 Fatty Acid):ti,ab,kw OR (Acid, Omega-3 Fatty):ti,ab,kw OR (Fatty Acid, Omega-3):ti,ab,kw OR (Omega 3 Fatty Acid):ti,ab,kw OR (Omega-3 Fatty Acids):ti,ab,kw OR (n-3 Oil):ti,ab,kw OR (n 3 Oil):ti,ab,kw OR (Oil, n-3):ti,ab,kw OR (n3 Oil):ti,ab,kw OR (Oil, n3):ti,ab,kw OR (n-3 Fatty Acids):ti,ab,kw OR (n 3 Fatty Acids):ti,ab,kw OR (n-3 Polyunsaturated Fatty Acid):ti,ab,kw OR (n 3 Polyunsaturated Fatty Acid):ti,ab,kw OR (n-3 PUFA):ti,ab,kw OR (n 3 PUFA):ti,ab,kw OR (PUFA, n-3):ti,ab,kw OR (n3 Fatty Acid):ti,ab,kw OR (Fatty Acid, n3):ti,ab,kw OR (n3 PUFA):ti,ab,kw OR (PUFA, n3):ti,ab,kw OR (n3 Polyunsaturated Fatty Acid):ti,ab,kw OR (n3 Oils):ti,ab,kw OR (Omega 3 Fatty Acids):ti,ab,kw OR (n-3 Oils):ti,ab,kw OR (n 3 Oils):ti,ab,kw
6. #4 AND #5
7. #3 AND #6 (119 results)

Text S4 Search strategy

Database: **Web of Science** from inception to Present> (Search date: 01, 08, 2025)

Search Strategy:

1. TS=(stomach neoplasms OR Neoplasm, Stomach OR Stomach Neoplasm OR Gastric Neoplasms OR Gastric Neoplasm OR Neoplasm, Gastric OR Neoplasms, Gastric OR Neoplasms, Stomach OR Cancer of Stomach OR Stomach Cancers OR Cancer of the Stomach OR Gastric Cancer OR Cancer, Gastric OR Cancers, Gastric OR Gastric Cancers OR Stomach Cancer OR Cancers, Stomach OR Cancer, Stomach OR Gastric Cancer, Familial Diffuse)
2. TS=(Fatty Acids, Omega-3 OR N-3 Fatty Acid OR Acid, N-3 Fatty OR Fatty Acid, N-3 OR N 3 Fatty Acid OR Omega-3 Fatty Acid OR Acid, Omega-3 Fatty OR Fatty Acid, Omega-3 OR Omega 3 Fatty Acid OR Omega-3 Fatty Acids OR n-3 Oil OR n 3 Oil OR Oil, n-3 OR n3 Oil OR Oil, n3 OR n-3 Fatty Acids OR n 3 Fatty Acids OR n-3 Polyunsaturated Fatty Acid OR n 3 Polyunsaturated Fatty Acid OR n-3 PUFA OR n 3 PUFA OR PUFA, n-3 OR n3 Fatty Acid OR Fatty Acid, n3 OR n3 PUFA OR PUFA, n3 OR n3 Polyunsaturated Fatty Acid OR n3 Oils OR Omega 3 Fatty Acids OR n-3 Oils OR n 3 Oils)
3. #1 AND #2 (753 results)
4. Forest Plot of total postoperative complications


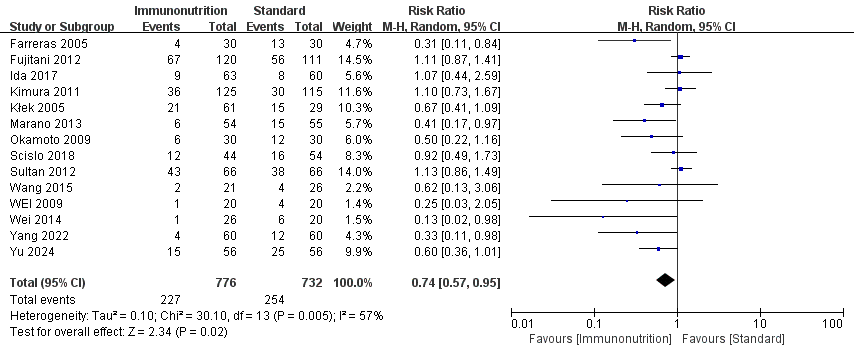


1. Funnel Plot of total postoperative complications


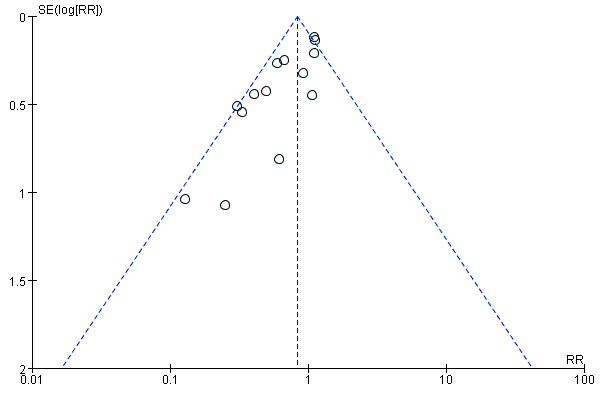


1. Forest Plot of CD4 lymphocytes


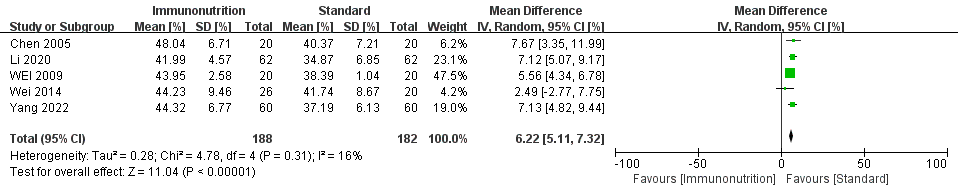


1. Forest Plot of CD4/CD8


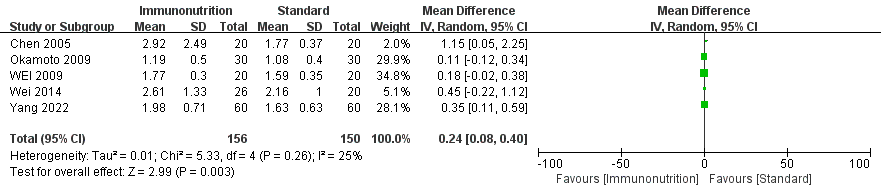


1. Forest Plot of CD8 lymphocytes


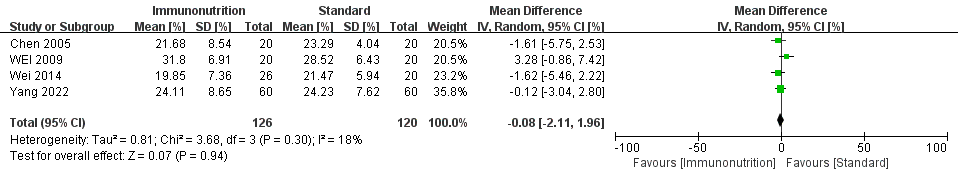


1. Forest Plot of total lymphocytes


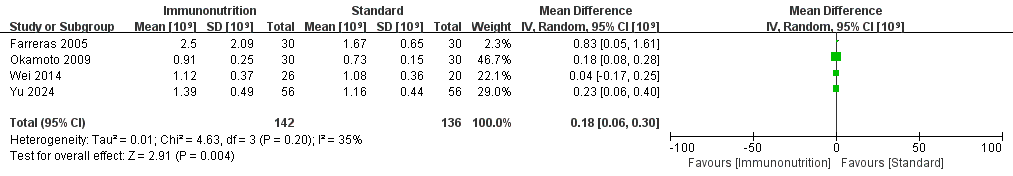


1. Forest Plot of IgA


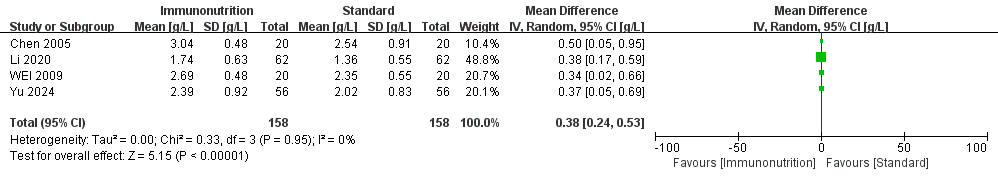


1. Forest Plot of IgG


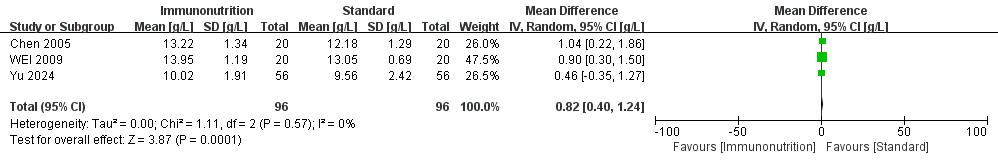


1. Forest Plot of IgM


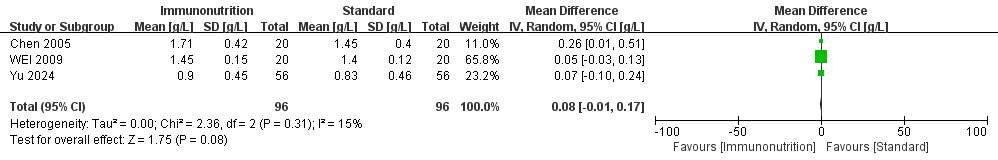


1. Forest Plot of transferrin


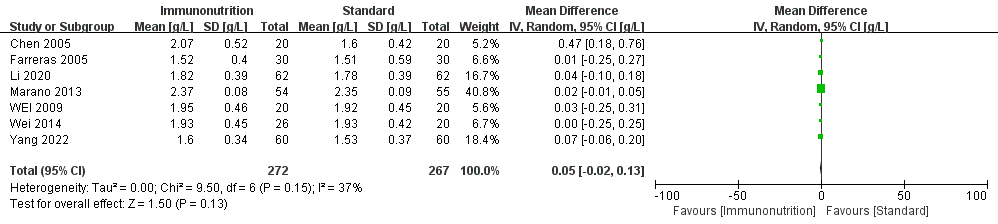


1. Forest Plot of albumin


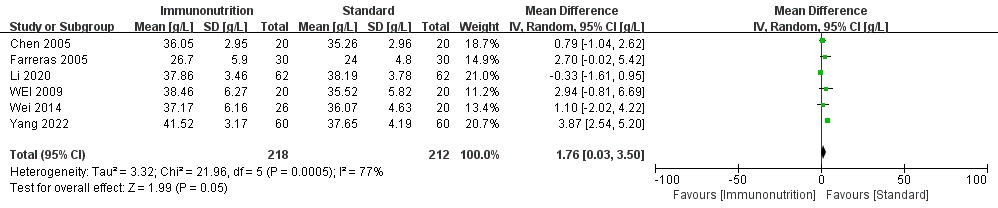


1. Forest Plot of prealbumin


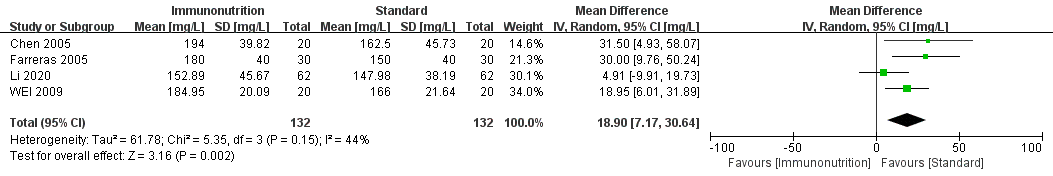


1. Forest Plot of IL-6


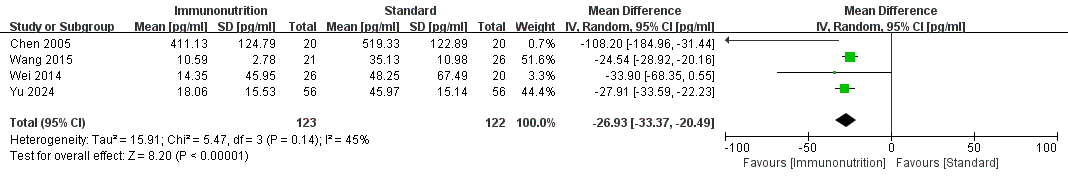


1. Forest Plot of TNF-α


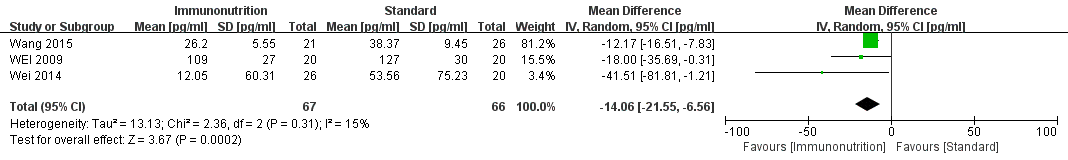


1. Forest Plot of CRP


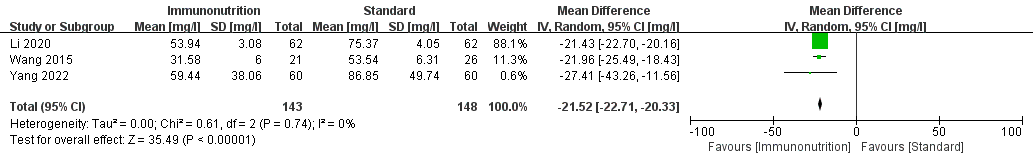


1. Forest Plot of time to first flatus


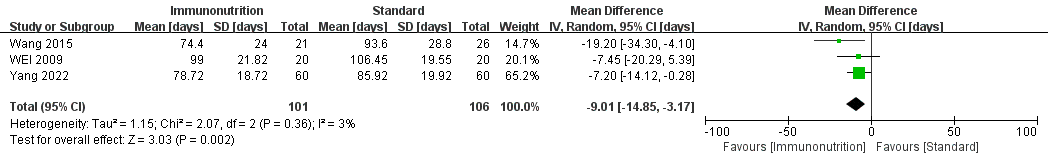


1. Forest Plot of length of hospital stay


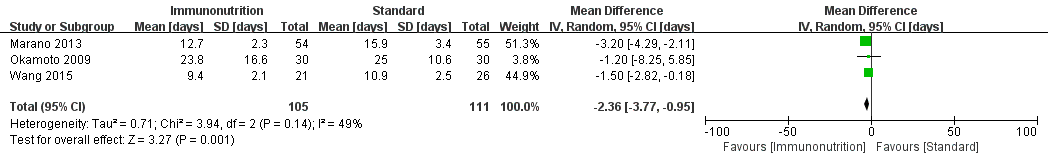


1. Forest Plot for subgroup analysis of total postoperative complications according to intervention timing.


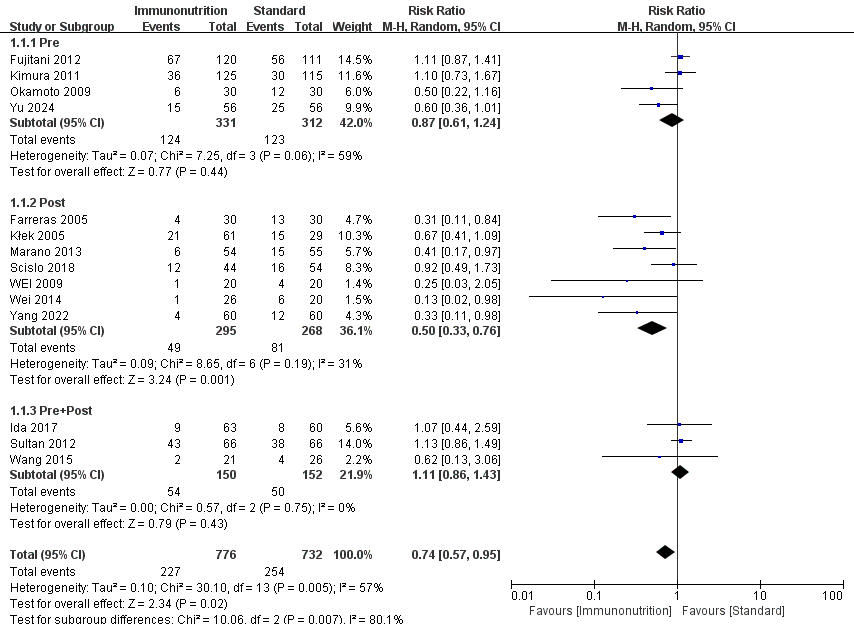


1. Forest Plot for subgroup analysis of total postoperative complications according to **type of immunonutrition regimen**.


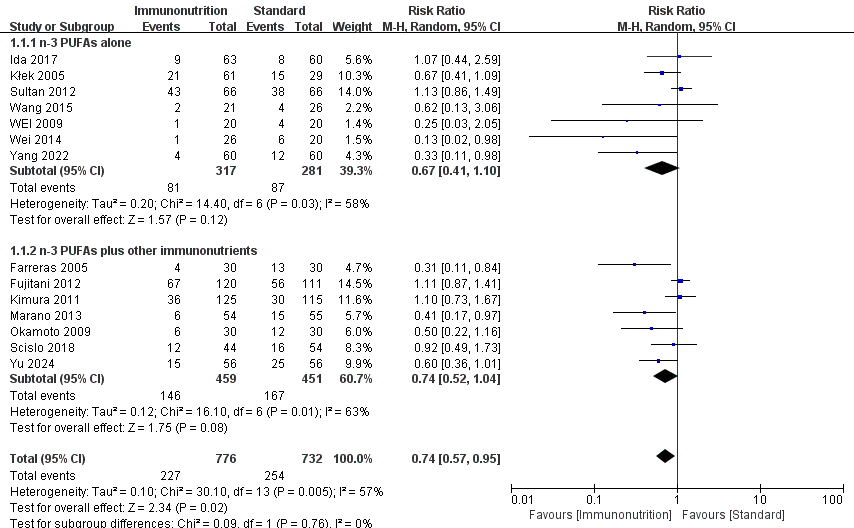


1. Forest Plot for subgroup analysis of total postoperative complications according to specific immunonutrient composition.


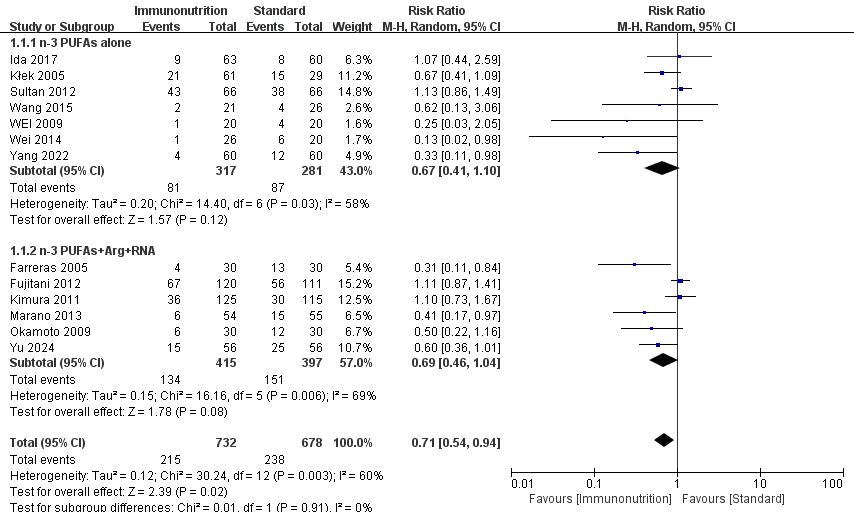


1. Forest Plot for subgroup analysis of total postoperative complications according to duration of postoperative administration.


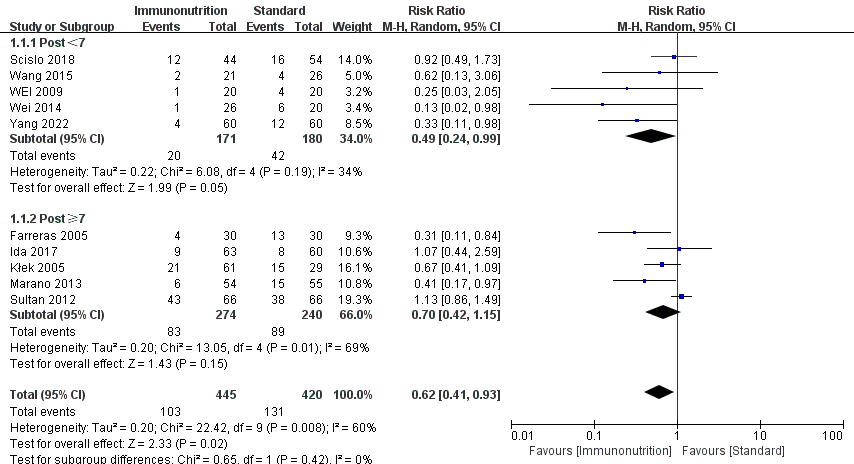


1. Forest Plot for subgroup analysis of total postoperative complications according to intervention method.


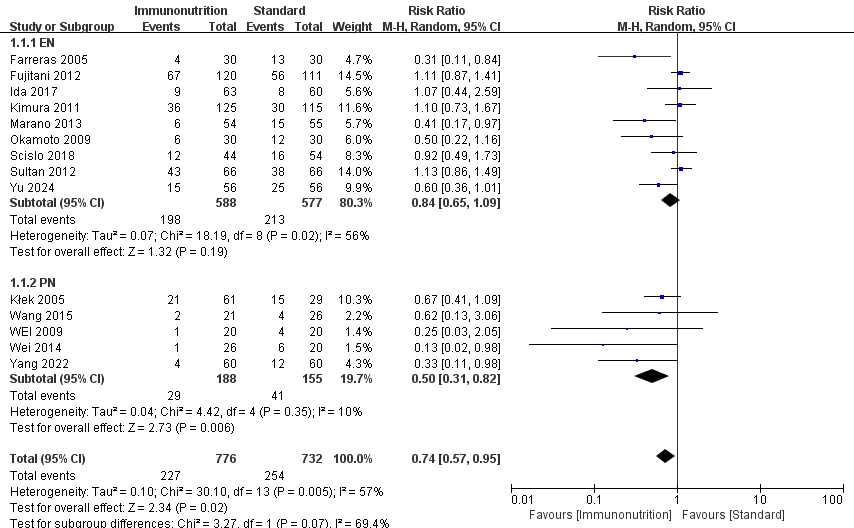

Supplement: Supplementary file 1 [file Table_1.docx]
